# CCR9-CCL25 interactions promote cisplatin resistance in breast cancer cell through Akt activation in a PI3K-dependent and FAK-independent fashion

**DOI:** 10.1186/1477-7819-9-46

**Published:** 2011-05-03

**Authors:** Crystal Johnson-Holiday, Rajesh Singh, Erica L Johnson, William E Grizzle, James W Lillard, Shailesh Singh

**Affiliations:** 1 Department of Microbiology, Biochemistry and Immunology, Morehouse School of Medicine, 720 Westview Drive SW, Atlanta, GA 30310 USA; 2 Department of Pathology, University of Alabama at Birmingham, West Pavilion P220 619 South 19th Street, Birmingham, AL 35233 USA

**Keywords:** chemokine, breast cancer, CCR9, CCL25, cisplatin resistance

## Abstract

**Background:**

Chemotherapy heavily relies on apoptosis to kill breast cancer (BrCa) cells. Many breast tumors respond to chemotherapy, but cells that survive this initial response gain resistance to subsequent treatments. This leads to aggressive cell variants with an enhanced ability to migrate, invade and survive at secondary sites. Metastasis and chemoresistance are responsible for most cancer-related deaths; hence, therapies designed to minimize both are greatly needed. We have recently shown that CCR9-CCL25 interactions promote BrCa cell migration and invasion, while others have shown that this axis play important role in T cell survival. In this study we have shown potential role of CCR9-CCL25 axis in breast cancer cell survival and therapeutic efficacy of cisplatin.

**Methods:**

Bromodeoxyuridine (BrdU) incorporation, Vybrant apoptosis and TUNEL assays were performed to ascertain the role of CCR9-CCL25 axis in cisplatin-induced apoptosis of BrCa cells. Fast Activated Cell-based ELISA (FACE) assay was used to quantify *In situ *activation of PI3K^p85^, Akt^Ser473^, GSK-3β^Ser9 ^and FKHR^Thr24 ^in breast cancer cells with or without cisplatin treatment in presence or absence of CCL25.

**Results:**

CCR9-CCL25 axis provides survival advantage to BrCa cells and inhibits cisplatin-induced apoptosis in a PI3K-dependent and focal adhesion kinase (FAK)-independent fashion. Furthermore, CCR9-CCL25 axis activates cell-survival signals through Akt and subsequent glycogen synthase kinase-3 beta (GSK-3β) and forkhead in human rhabdomyosarcoma (FKHR) inactivation. These results show that CCR9-CCL25 axis play important role in BrCa cell survival and low chemotherapeutic efficacy of cisplatin primarily through PI3K/Akt dependent fashion.

## Introduction

Breast cancer (BrCa) is second leading cause of cancer related deaths among women after lung cancer [1]. It is estimated that 207,090 women will be diagnosed with BrCa in 2010 and 39,480 will die from this disease in United States [1]. Cisplatin and its analogues have been widely used to treat human cancers including BrCa [2]. Unfortunately, many tumors become resistant to cisplatin, which is characterized by decreased susceptibility to apoptosis. Cancer cells develop resistance to chemotherapy primarily by inactivating apoptotic factors and/or enhancing cell survival pathways [3].

Chemokines are a group of small proteins (8 - 10 kDa), structurally related molecules that regulate trafficking of lymphocytes through interactions with a subset of seven-transmembrane G-protein coupled receptors [4]. To this end, we have recently shown significantly higher expression of CCR9 in ovarian and prostate cancer cell lines and tumors [5,6]. CCR9-CCL25 interactions known to support T cells survival during thymic maturation by inhibiting apoptosis through Akt/protein kinase B activation, which is PI3K- and Gα_i _protein-dependent [7,8]. Indeed, the PI3K/Akt anti-apoptotic and survival pathway plays a crucial role in cisplatin resistance [9]. FAK has also been shown to support anti-apoptotic mechanisms through Akt signaling [10]. Chemokine receptors may also aggregate with integrins following stimulation to promote FAK phosphorylation.

This study investigates the role of CCR9-CCL25 interactions and requirement for PI3K and FAK activation in BrCa cell survival and cisplatin resistance. We show for the first time that CCR9-CCL25 interactions provide protection from cisplatin-induced BrCa cell death. We also show that CCR9-CCL25 interaction promotes cell proliferation and upregulated anti-apoptotic signaling, which is mediated by the PI3K/Akt survival pathway, independent of FAK. These studies suggest that the expression of functional CCR9 may facilitate BrCa cell survival and low chemotherapeutic response.

## Materials and methods

### Cell culture

Human BrCa cell lines, MDA-MB-231 and MCF-7, were obtained from ATCC. These cells were cultured in RPMI-1640 media (Mediatech, Inc.) supplemented with 10% fetal bovine serum (FBS; Sigma) at 37°C with 5% CO_2_. Prior to each experiment, cells were cultured for 24 hours in RPMI 1640 with 1% FBS to serum starve the cells.

### Bromodeoxyuridine incorporation assay

BrCa cells (10^5^) were cultured alone or with 100 ng/mL CCL25 + 1 μg/mL of isotype control antibody or 100 ng/mL CCL25 + 1 μg/mL anti-CCR9 antibody (clone 112509, R&D Systems) for 24 hours with 0, 0.1, 1, 2, 5, 10, 25, or 50 μg/mL of cisplatin. Incorporation of bromodeoxyuridine (BrdU) into newly synthesized DNA permits indirect detection of rapidly proliferating cells. Hence, this assay was used according to manufacturer's instructions to estimate BrCa cell growth. Briefly, cells were treated with BrdU for 18 hours at 37°C. Media containing labelling solution was removed and cells were washed twice with media containing 10% serum. BrCa cells were fixed with 200 μL of fixative solution for 30 minutes at ~25°C and washed as before. Next, cells were incubated with 100 μL of nuclease solution for 30 minutes at 37°C and washed 3 times. Subsequently, 100 μL of anti-BrdU antibody was added, incubated for 30 minutes at 37°C, and washed 3 times. BrdU incorporation by BrCa cells was detected by peroxidase substrate reaction. After the extinction of this reaction, the samples were measured in a micro plate reader at 405 nm with a reference wavelength at approximately 490 nm.

### Vybrant apoptosis assay

BrCa cells were cultured in RPMI-1640 media with 5 μg/ml of cisplatin (IC^50^), along with no additions or 100 ng/mL of CCL25 or 1 μg/mL of anti-CCR9 Ab or 100 ng/mL of CCL25 plus 1 μg/mL of anti-CCR9 Ab for 24 hours. The cells were harvested and washed in ice-cold PBS. Cells (10^5 ^cells/mL) were stained with Annexin-V and propidium iodide (PI) using the Vybrant apoptosis assay Kit #3 (Invitrogen) according to manufacturer's protocol. The stained cells were analyzed by flow cytometry using UV/488 nm dual excitation and measuring the fluorescence emission at approximately 530 nm and 575 nm.

### Terminal Transferase dUTP Nick End Labeling (TUNEL) Assay

BrCa cells were cultured with 0 or 5 μg/ml (IC^50^) of cisplatin, along with no additions or 100 ng/mL of CCL25 plus 1 μg/mL of anti-CCR9 or isotype control antibodies for 24 hours. Apoptosis was measured by TUNEL assay (Millipore) according to the manufacturer's instructions. Briefly, following treatment the cells were fixed with 4% paraformaldehyde in 0.1 M NaH_2_PO_4_, pH 7.4 for 15 minutes. After washing in PBS three times, the cells were incubated with 0.05% Tween-20 in PBS for 15 minutes. After washing in PBS, the cells were incubated with TdT end-labelling cocktail for 60 minutes. Termination buffer was added to stop the reaction. After washing 4 times in PBS, cells were blocked for 20 minutes and stained with avidin-fluorescein isothiocyanate (FITC) solution for 30 minutes. After washing with PBS 3 times, fluorescence plate reader quantified the fluorescence of TUNEL positive cells.

### Fast activated cell-based ELISA (FACE)

The levels of total and active (phosphorylated) PI3K^p85^, Akt^Ser473^, GSK-3β^Ser9^, and FKHR^Thr24 ^were quantified using Fast Activated Cell-based ELISA (FACE) assays (Active Motif) according to the manufacturer's instructions. Briefly, cells were cultured in 96-well plates one day prior to manipulation. BrCa cells were cultured in serum-free media with 0 or 5 μg/ml of cisplatin or 100 ng/ml of CCL25 or 5 μg/mL of cisplatin with 100 ng/mL of CCL25 for 24 hours. In addition, cells were treated with or without kinase inhibitors of PI3K (wortmannin; Sigma) and FAK (PF-573, 228;Pfizer).

### Statistics

The data were compared using a two-tailed Student's t test and expressed as the mean ± SE. The results were analyzed using the Statview II program (Abacus Concepts, Inc., Berkeley CA) and were labeled statistically significant if *p *values were < 0.01.

## Results

### Effects of CCL25 on cisplatin-induced growth inhibition

**Figure 1:**
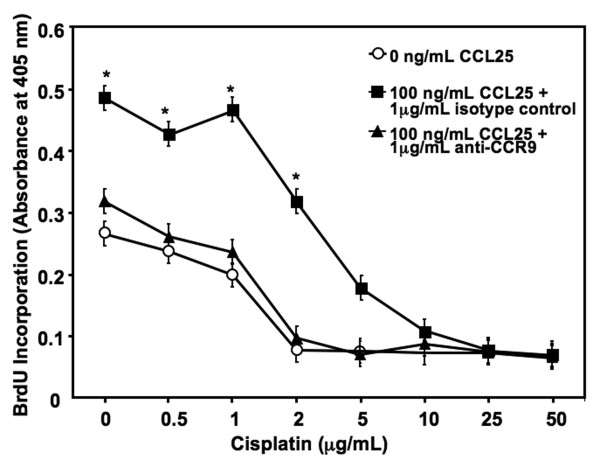
**CCL25 inhibits cisplatin-induced reductions in cell growth**. MDA-MB-231 cells were cultured with 0 or 100 ng/ml of CCL25 plus isotype control or anti-CCR9 Ab (1 μg/mL) for 24 hours, along with increasing concentrations of cisplatin (0-50 μg/mL). Cell proliferation was determined by BrdU incorporation in triplicate and was repeated 3 times. Asterisks (*) indicate significant differences (*p *< 0.01) between CCL25-treated and untreated BrCa cells.

CCL25 significantly enhanced the growth of MDA-MB-231 cells in comparison to untreated (cisplatin-free BrCa) cells (Figure 1). While cisplatin concentrations of ≤ 1 μg/mL had no major effect on either CCL25-treated or untreated BrCa cell growth, 2 μg/mL of cisplatin reduced cell proliferation of untreated, but not CCL25-treated cells. In fact, CCL25 significantly protected cisplatin (< 5 μg/mL)-mediated growth inhibition. As the concentration of cisplatin reached ≥ 10 μg/mL, the effect of CCL25-mediated protection of cisplatin-induced BrCa cell growth inhibition dissipated. Importantly, the significant increases of BrCa cell proliferation and cisplatin-dependent growth inhibition caused by CCL25 treatment was abrogated by CCR9 blockade.

### CCL25-induced survival and cisplatin resistance of breast cancer cells

**Figure 2:**
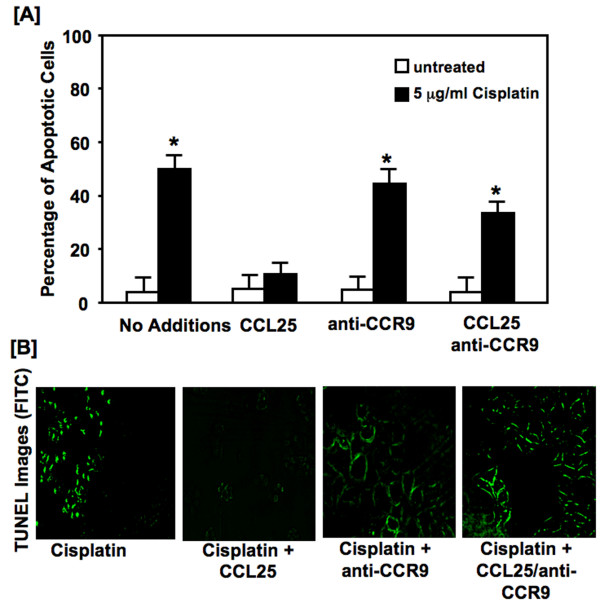
**Cisplatin-induced apoptosis**. *Panel A: *MDA-MB-231 cells were cultured for 24 hours with 5.0 μg/ml of cisplatin with or without CCL25 (100 ng/mL) plus 1 μg/mL of anti-human CCR9 or isotype controls. Cells were stained with annexin V and propidium iodide (PI). Analysis by flow cytometry of the stained cells distinguished apoptotic (annexin V positive) cells from viable (no fluorescence) and necrotic (PI positive) cells. Asterisks (*) indicate significant differences (p < 0.01) between CCL25-treated and untreated BrCa cells. *Panel B: *MDA-MB-231 cells were cultured for 24 hours with 5.0 μg/mL cisplatin or with 0 or 100 ng/ml of CCL25 plus anti-human CCR9 or isotype control Abs (1 μg/mL). Detection of apoptotic cells was carried out using the terminal deoxynucleotidyl transferase-mediated dUTP nick-end labeling (TUNEL) method. Apoptotic cells exhibited nuclear green fluorescence with a standard fluorescence filter set (520 ± 20 nm).

Cisplatin treatment resulted in a ~5 fold increase in apoptotic cells, relative to the untreated cells (Figure 2A). However, CCL25 significantly decreased the percentage of cisplatin-treated apoptotic cells, compared to untreated or anti-CCR9 Ab treated cells. As with growth inhibition, the decrease in the percentage of cisplatin-induced apoptotic cells (or increase in cell survival) afforded by CCL25 was abolished by CCR9 inhibition. Apoptosis was also assessed under the same conditions by TUNEL analysis (Figure 2B). BrCa cells treated with cisplatin showed an increase in apoptosis as compare to the untreated cells. However, similar cisplatin-treated cells co-cultured with CCL25 displayed significantly low apoptotic or TUNEL-positive events, which was abrogated after CCR9 blockade.

### CCR9-CCL25-interactions lead to PI3K p85, Akt, GSK-3β and FKHR activation

**Figure 3:**
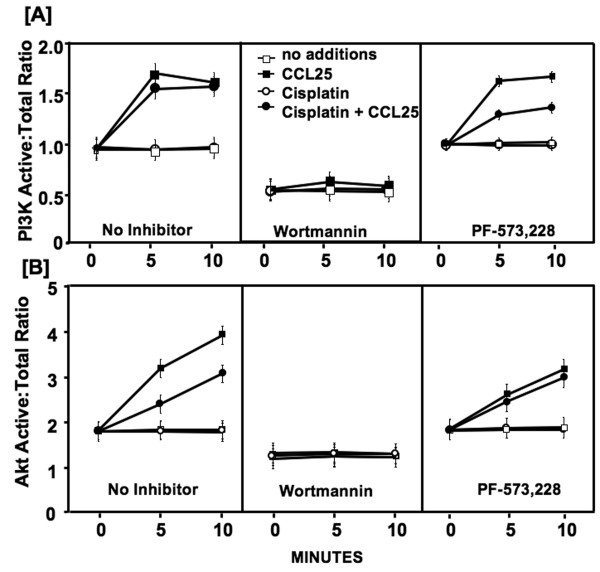
**PI3K and Akt activation by CCR9-CCL25 interactions**. MDA-MB-231 cells were tested for their ability to activate PI3K and Akt following treatment with CCL25, cisplatin and specific kinase inhibitors (wortmannin and PF-573, 228). *In situ *total and active (phosphorylated) PI3K and Akt levels were quantified by Fast Activated Cell-based ELISA (FACE) assay before (0 minutes) or after (5 or 10 minutes) CCL25 stimulation in the presence of cisplatin and kinase inhibitors.

To identify the cellular signals involved in cisplatin resistance mediated by CCR9-CCL25 interaction, *in situ *PI3K p85, Akt, GSK-3β, and FKHR phosphorylation levels were quantified by FACE assay. Cisplatin alone had no significant effect on PI3K activity in comparison to untreated cells (Figure 3A). CCL25 induced a rapid increase in PI3Kp85 activation after 5 minutes, which continued through 10 minutes in the presence or absence of cisplatin. As expected, treatment with wortmannin, a PI3K inhibitor prevented CCL25-dependent increases in PI3K activity in all samples. In contrast, FAK inhibition had no effect on CCL25-mediated PI3Kp85 phosphorylation. Interestingly, the immediate increase in PI3K activation by CCL25 and cisplatin co-cultured cells was moderately attenuated by FAK inhibition.

The active (phosphorylated) to total Akt protein ratio in BrCa cells not treated with CCL25 was approximately 2:1 (Figure 3B). Treatment with cisplatin alone had no effect on Akt activity. There was a significant increase in Akt phosphorylation ≥ 5 minutes after CCL25 treatment. This CCL25-mediated Akt activity was partially reduced by cisplatin co-culture. Wortmannin treated cells were non-responsive to CCL25 stimulation, while FAK inhibition had no effect on CCL25-mediated Akt phosphorylation.

**Figure 4:**
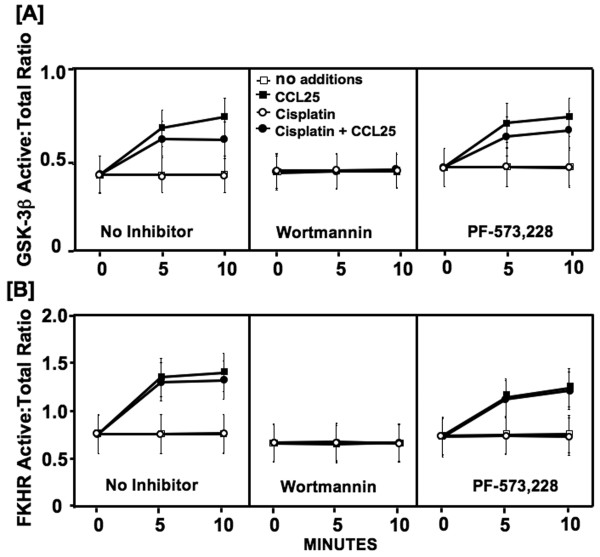
**GSK-3β and FKHR phosphorylation following CCL25 treatment**. MDA-MB-231 cells were tested for their ability to phosphorylate GSK-3β and FKHR following treatment with CCL25, cisplatin and specific-kinase inhibitors (wortmannin and PF-573, 228). *In situ *total and active (phosphorylated) GSK-3β and FKHR levels were quantified by Fast Activated Cell-based ELISA (FACE) assay before (0 minutes) or after (5 or 10 minutes) CCL25 stimulation in the presence of cisplatin and kinase inhibitors.

Activated Akt phosphorylates and inactivates the anti-proliferative effects of GSK-3β. In the presence or absence of cisplatin, CCL25 significantly increased GSK-3β phosphorylation ≥ 5 minutes after treatment (Figure 4A). Wortmannin pre-treatment completely removed this effect. However, FAK inhibition did not significantly alter GSK-3β phosphorylation by CCL25 with or without cisplatin co-culture.

Akt also inactivates FKHR through phosphorylation, which leads to anti-apoptotic events or cell survival. CCL25 induced a significant and rapid increase in FKHR phosphorylation levels in either cisplatin-free or -treated BrCa cells (Figure 4B). Wortmannin treatment eliminated this effect. However, FAK-inhibition had no effect on CCL25-mediated FKHR phosphorylation.

## Discussion

According to the American Cancer Society, in 2010, 207,090 women will be diagnosed with BrCa and about 39,840 women will die from the disease [1]. Although death rates have been declining since 1990, BrCa mortality is high among African American women [11]. Many of these deaths are due to chemo-resistance, which is a common problem in the treatment of BrCa [12,13]. Cisplatin and its analogues have been widely used for treatment of human cancers, including advanced BrCa [14-16]. However, resistance to cisplatin represents a major obstacle in the effective management of metastatic BrCa [17]. The balance between survival and apoptotic signals in cancer cells determine the sensitivity to chemotherapy and cancer cells develop resistance to chemotherapies by inactivating apoptotic factors and enhancing survival pathways [18]. However, the factors that promote these remain incompletely understood.

Chemokines direct the migration of leukocytes as well as cancer cells [19,20] and they play a pivotal role in cell survival [21]. Interactions between CXCR4 and its ligand, CXCL12, promote the survival of breast [4], pancreatic cancers [22], and melanoma [23]. CCR9-CCL25 interactions also potentiate anti-apoptotic signaling to immature, or double-positive T cells [24], and function to retain single positive cells in the thymus until they are fully mature and ready for export [7].

We have demonstrated that CCR9 is significantly expressed by ovarian and prostate cells and play important role in cell migration and invasion [25,26]. Here we show that CCR9 also supports BrCa cell growth as well as cell survival or resistance to cisplatin. CCL25 significantly increases BrCa cell proliferation and cisplatin resistance in a CCR9-dependent fashion.

It has been previously shown that CCR9 signaling plays a role in immature T cell survival through PI3K and Gα_i _protein-dependent activation of Akt/protein kinase B [27]. Downstream PI3K mediators directly phosphorylate and activate Akt [28,29]. PI3K/Akt signaling pathways are also frequently disturbed in many human cancers and evidence suggests that chemokine receptor signaling activates Akt [28,30]. Akt modulates the function of numerous substrates involved in the regulation of cell survival, cell cycle progression and cellular growth. The PI3K/Akt pathway is also involved in chemoresistance to cisplatin [9]. Phosphorylated Akt promotes survival by phosphorylating and inactivating pro-apoptotic factors, such as GSK-3β and or FKHR. GSK-3β inhibition by Akt prevents phosphorylation of β-catenin, which impedes its degradation; hence, it is translocated to the nucleus. Once in the nucleus, β-catenin combines with different transcription factors to induce the expression of several genes, such as cyclin D1 [29]. Hence, Akt-mediated phosphorylation of GSK-3β prevents the accumulation of cyclin D1, which is needed to support cell cycle progression. FKHR transactivates the expression of death activating proteins, such as Fas ligand (FasL), Bim, and Bcl-6. Phosphorylating FKHR1 at its threonine and serine residues prevents its translocation to the nucleus and any associated gene transcription [31].

Our studies strongly support the hypothesis that CCR9-CCL25 signaling enhances BrCa cell growth and survival. Specifically, we show that CCL25 induces the activation of the PI3K/Akt pathway and phosphorylation of its downstream mediators, e.g., GSK-3β and FKHR. PI3K inhibition completely abrogated CCL25-mediated and CCR9-dependent cisplatin resistance, Akt, GSK-3β, and FKHR phosphorylation. However, it was also plausible that other Akt-activation pathways supported BrCa cell survival following cisplatin (and CCL25) treatment. In this regard, chemokine-chemokine receptor interactions also support integrin clustering to potentially activate FAK, which is a protein tyrosine kinase involved in cell proliferation, migration and survival [32]. Activated FAK also interacts with PI3K through integrin clustering [33]. However, our findings show that FAK is not involved in CCL25-mediated and CCR9-dependent Akt activation or subsequent GSK-3β and FKHR phosphorylation.

In support of our findings, a recent study has shown that the PI3K/Akt signaling pathway is an important event downstream of amphiregulin for the development of cisplatin resistance in BrCa cells [34]. Taken together, these results suggest that CCL25 treatment induces BrCa cell survival and cisplatin resistance. We also show that CCR9-dependent anti-apoptotic signaling involves the PI3K/Akt pathway and phosphorylation of its downstream mediators, GSK-3β and FKHR - through CCR9 and PI3K/Akt, but independent of FAK, supporting our hypothesis that CCR9-CCL25 interaction promotes BrCa cell survival and resistance to cisplatin.

## Conclusion

These results suggest that CCR9-CCL25 axis play significant role in BrCa cell survival and cisplatin resistance primarily through PI3K/Akt dependent fashion.

## Competing interests

The authors declare that they have no competing interests.

## Authors' contributions

CJH conducted the experiments, analyzed data and drafted the manuscript. RS also analyzed the data and assisted with manuscript preparation. ELJ, WEG, and SS assisted with experiments and manuscript preparation. JWL conceptualized, edited, and revised the manuscript. All authors have read and approved the final manuscript.
